# The *indica* nitrate reductase gene *OsNR2* allele enhances rice yield potential and nitrogen use efficiency

**DOI:** 10.1038/s41467-019-13110-8

**Published:** 2019-11-15

**Authors:** Zhenyu Gao, Yufeng Wang, Guang Chen, Anpeng Zhang, Shenglong Yang, Lianguang Shang, Danying Wang, Banpu Ruan, Chaolei Liu, Hongzhen Jiang, Guojun Dong, Li Zhu, Jiang Hu, Guangheng Zhang, Dali Zeng, Longbiao Guo, Guohua Xu, Sheng Teng, Nicholas P. Harberd, Qian Qian

**Affiliations:** 1 State Key Laboratory of Rice Biology, China National Rice Research Institute, Chinese Academy of Agricultural Sciences, Hangzhou, 310006 China; 2 Institute of Plant Physiology and Ecology, Shanghai Institutes for Biological Sciences, Chinese Academy of Sciences, Shanghai, 200032 China; 3 Lingnan Laboratory of Modern Agriculture, Genome Analysis Laboratory of the Ministry of Agriculture, Agricultural Genomics Institute at Shenzhen, Chinese Academy of Agricultural Sciences, Shenzhen, 518124 China; 4 State Key Laboratory of Crop Genetics and Germplasm Enhancement, College of Resources and Environmental Sciences, Nanjing Agricultural University, Nanjing, 210095 China; 5 Department of Plant Sciences, University of Oxford, South Parks Road, Oxford, OX1 3RB UK

**Keywords:** Agricultural genetics, Quantitative trait, Natural variation in plants, Transgenic plants

## Abstract

The *indica* and *japonica* rice (*Oryza sativa*) subspecies differ in nitrate (NO_3_^−^) assimilation capacity and nitrogen (N) use efficiency (NUE). Here, we show that a major component of this difference is conferred by allelic variation at *OsNR2*, a gene encoding a NADH/NADPH-dependent NO_3_^−^ reductase (NR). Selection-driven allelic divergence has resulted in variant *indica* and *japonica OsNR2* alleles encoding structurally distinct OsNR2 proteins, with *indica* OsNR2 exhibiting greater NR activity. *Indica OsNR2* also promotes NO_3_^−^ uptake via feed-forward interaction with *OsNRT1.1B*, a gene encoding a NO_3_^−^ uptake transporter. These properties enable *indica OsNR2* to confer increased effective tiller number, grain yield and NUE on *japonica* rice, effects enhanced by interaction with an additionally introgressed *indica OsNRT1.1B* allele. In consequence, *indica OsNR2* provides an important breeding resource for the sustainable increases in *japonica* rice yields necessary for future global food security.

## Abstract (web summary)

*Indica* rice has higher nitrate assimilation and nitrogen use efficiency (NUE) than *japonica* rice, but the mechanism is unclear. Here, the authors reveal that the difference is partly due to allelic variation of a nitrate reductase encoding gene and this *indica* allele can increase yield potential and NUE.

## Introduction

Two distinct cultivated *indica* and *japonica* subspecies arose during Asian rice domestication^1,2^. *Indica*- and *japonica*-type cultivars can be reliably distinguished using characteristic traits^3–5^, among which is relative resistance to toxic chlorate (ClO_3_^−^) ion. However, while the genes underlying several of these distinguishing traits have been mapped or molecularly cloned^6–11^, the molecular and evolutionary causes of differential rice ClO_3_^−^ resistance^12^ remain unclear. ClO_3_^−^ is relatively toxic to rice and other plants because it is a NO_3_^−^ analog^13^. Soil NO_3_^−^ is absorbed through specific root uptake transporters^14^, and subsequently reduced to nitrite (NO_2_^−^) by the NR enzyme of NO_3_^-^ assimilation^15^. The ClO_3_^−^ is absorbed by NO_3_^−^ uptake transporters, and then reduced by NR to the toxic chlorite (ClO_2_^−^), which inhibits plant growth. Accordingly, mutants deficient in NO_3_^−^ uptake transporter or NR function are relatively ClO_3_^−^ resistant^16,17^.

Rice is mostly cultivated in anaerobic paddy field soils, where ammonium (NH_4_^+^) rather than NO_3_^−^ predominates as N source. Nevertheless, specialized aerenchyma rice root cells transfer oxygen from shoot to root, causing release into the rhizosphere. The resultant enhancement of soil bacterial conversion of NH_4_^+^ to NO_3_^−^ (nitrification)^18^ provides 15–40% of paddy field grown rice N uptake^19^. The efficiency of NO_3_^−^ uptake and assimilation is therefore a key effector for paddy-grown rice productivity, and variation in ClO_3_^−^ resistance is a valuable proxy indicator for relative rice NO_3_^−^ assimilation efficiencies.

Here, we identify major quantitative trait loci (QTLs) conferring the ClO_3_^−^ resistance differences between the *indica* (variety 9311) and *japonica* (Nipponbare) rice subspecies. Fine mapping, molecular cloning, and further analyses show one of these QTLs (*qCR2*) to encode the NAD(P)H-dependent nitrate reductase (NR) OsNR2. We also show that a key arginine residue (Arg_783_) in the NAD(P) binding domain of the 9311 (*indica*) OsNR2 confers a specific NR activity greater than that of the Nipponare (*japonica*) OsNR2, in which a Trp residue is substituted for Arg_783_. Further phylogenetic and evolutionary analyses suggest that allelic divergence at *OsNR2* has been driven by directional selection on both *indica* and *japonica* alleles. Finally, we show that the *indica OsNR2* allele confers superior grain yield and NUE (versus the *japonica* allele), in part via feed-forward interaction with *OsNRT1.1B*, a gene coding for a NO_3_^−^ uptake transporter^20^.

## Results

### *OsNR2* is a major determinant of rice ClO_3_^−^ resistance

**Fig. 1:**
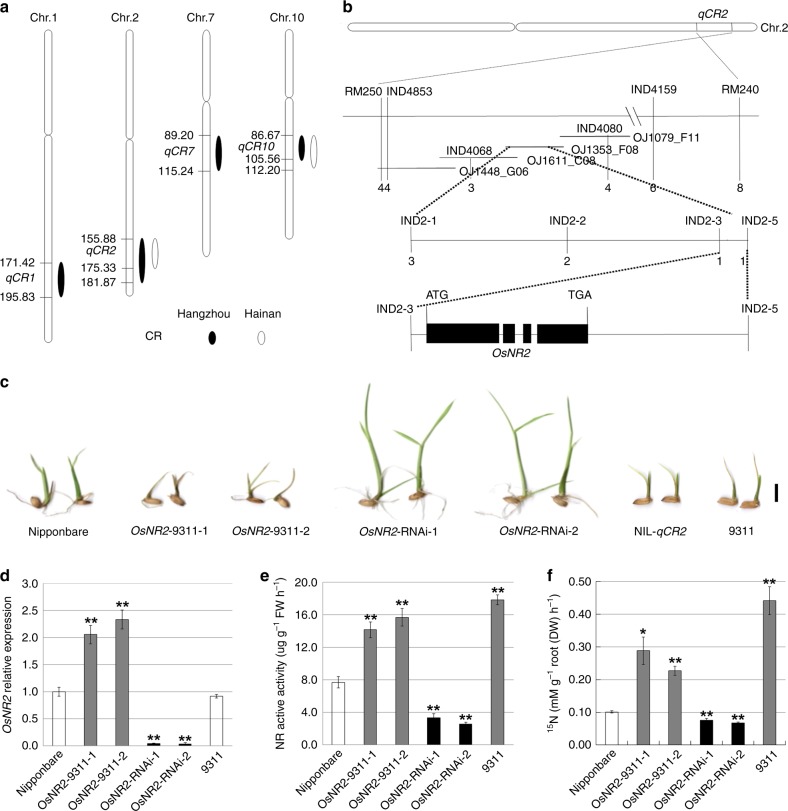
*OsNR2* allelic variation confers the *qCR2*, and affects NR active activity and ^15^NO_3_- uptake. **a** ClO_3_^−^ resistance (CR) QTL analysis of RILs from an *indica* (9311) × *japonica* (Nipponbare) cross, performed on seedlings germinated from seeds harvested in Hangzhou or Hainan (see Methods). Major QTLs are shown, with numbers indicating genetic map position (cM) on each chromosome. **b** Fine mapping of *qCR2* with a residual heterozygote line (RHL) F_2_ population. Using a panel of linked markers (Supplementary Table 2), *qCR2* was pin-pointed to a 6.4 kb region (Chr.2, between markers IND2-3 and IND2-5) containing *OsNR2*. Numbers of recombinants between each marker and *qCR2* are shown. *OsNR2* structure is shown, black boxes represent exons. **c** Relative seedling vigor indicates degree of ClO_3_^−^ resistance (Nipponbare harboring constructs for expression of the 9311 *OsNR2* allele driven by the 9311 *OsNR2* promoter (*OsNR2*-9311-1 and *OsNR2*-9311-2) or of *OsNR2* RNAi (*OsNR2-*RNAi-1 and *OsNR2-*RNAi-2); see Supplementary Fig. 1). **d** Leaf *OsNR2* mRNA abundance, **e** leaf NR active activity, **f** ^15^NO_3_^−^ uptake activity of roots exposed to 1.25 mM ^15^NO_3_^−^. Value is mean ± s.d. (*n* = 3 for **d**–**f**). Error bar represents s.d. * and ** respectively indicate least significant differences at the 0.05 and 0.01 probability level compared with Nipponbare. The source data underlying Fig. 1d–f are provided as a Source Data file

Six major ClO_3_^−^ resistance QTLs were detected in analysis of recombinant inbred line (RIL) seedlings from a 9311 (*indica*) × Nipponbare (NPB, *japonica*) cross (Fig. 1a and Supplementary Table 1). Of these, the map positions of *qCR2* and *qCR10* correspond with previously identified ClO_3_^−^ resistance QTLs^21^, with *qCR10* reflecting variation at *OsNRT1.1B*, a gene encoding a NO_3_^-^ uptake transporter^20^. Because *qCR2* is a strong ClO_3_^−^ resistance QTL (with PVE of 8.0 in Hangzhou (HZ) and 23.8 in Hainan (HN), Supplementary Table 1), we delimited it to a single candidate *OsNR2* gene (see Methods, Fig. 1b and Supplementary Table 2). *OsNR2* encodes OsNR2, a NAD(P)H-dependent nitrate reductase (NR) (http://rice.plantbiology.msu.edu/) having dual ability to accept both NADPH and NADH as electron donors^22^. Expression of the 9311 *OsNR2* allele (driven by the 9311 *OsNR2* promoter) in Nipponbare increases ClO_3_^−^ sensitivity (Fig. 1c and Supplementary Fig. 1a), while reduction of *OsNR2* mRNA abundance increases Nipponbare ClO_3_^−^ resistance (Figs. 1c, 1d; Supplementary Fig. 1b), thus confirming that allelic variation at *OsNR2* is causal of *qCR2*.

A dose experiment for nitrate reductase activity in different concentration of nitrate and NADPH showed NR active activity of Nipponbare and 9311 were dependent on KNO_3_ concentration and NADPH concentration, respectively (Supplementary Fig. 2a, 2b). Therefore, 5 mM KNO_3_ and 0.2 mM NADPH were selected for NR activity assay. Km of NR active activity for NADPH and nitrate in Nipponbare were higher than those in 9311 (Supplementary Table 3), suggesting allelic variation at OsNR2 may affect binding ability with NADPH and NO_3_^−^. We also found that expression of 9311 *OsNR2* coding sequence or reduction in *OsNR2* mRNA abundance respectively caused increase or decrease in Nipponbare maximal and active NR activity (Fig. 1e, Supplementary Fig. 2c), changes in enzymatic activity that mirror the effect of allelic variation at *OsNR2* on relative ClO_3_^−^ resistance. In addition, expression of 9311 *OsNR2* coding sequence or reduction in *OsNR2* mRNA abundance caused intriguing parallel effects on NO_3_^−^ uptake capacity (Fig. 1f), with the 9311 *OsNR2* allele conferring relative increase in rate of NO_3_^−^ uptake.

### The Trp_779_ substitution reduces *japonica* OsNR2 activity

**Fig. 2:**
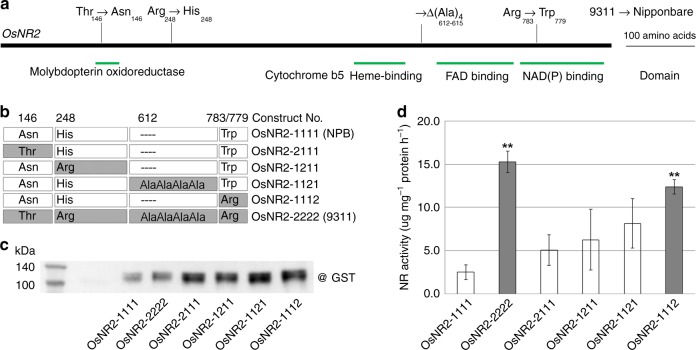
An Arg-Trp amino acid substitution reduces the specific activity of Nipponbare OsNR2. **a** Amino acid substitution and insertion differences between the 9311 and Nipponbare OsNR2 proteins. Locations of conserved functional domains are indicated by green bars. **b** Constructs expressing chimeric OsNR2 proteins of varying amino acid sequence. Fragments from the 9311 and Nipponbare OsNR2 coding regions were exchanged to generate constructs encoding a series of four different chimeric OsNR2 proteins, differing with respect to the amino acid residues variant between 9311 and Nipponbare OsNR2. These constructs were expressed in *E.coli*. **c** Detection with anti:GST antibody (Cat No: CW0084M, CWBIOtech, Beijing, China) of OsNR2-GST fusion proteins extracted from *E. coli* and purified on a GST-sefinose^TM^ column (constructs as shown in **b**), showing roughly equivalent abundance of OsNR2 proteins. **d** NR activity of purified extracts as in **b**, **c**. Value is mean ± s.d. (*n* = 3). Error bar represents s.d. ** indicates the least significant difference at 0.01 probability level compared with Nipponbare. The source data underlying **c** and **d** are provided as a Source Data file

Despite the differences in conferred ClO_3_^−^ resistance, *OsNR2* mRNA abundance does not detectably differ between 9311 and Nipponbare (Fig. 1d), suggesting that the 9311 OsNR2 protein is intrinsically more active than Nipponbare OsNR2. Accordingly, we found that 9311 and Nipponbare *OsNR2* protein-encoding regions differ by 3 nonsynonymous SNPs (conferring Thr_146_ to Asn_146_, Arg_248_ to His_248_ and Arg_783_ to Trp_779_ substitutions; 9311 to Nipponbare respectively) and by a 12-bp indel (conferring deletion of Ala_612-615_ from Nipponbare OsNR2; Fig. 2a and Supplementary Fig. 3). Because Thr_146_, Arg_248_ and Arg_783_ (as in 9311 OsNR2) are all strictly conserved in OsNR2 orthologues from related grass species (Supplementary Fig. 4), we determined the individual effects of these residues on the activity of *E.coli*-expressed OsNR2 (Fig. 2b). Using NADH as electron donor, no significant differrence was found between 9311 and Nipponbare OsNR2 protein (Supplementary Fig. 5). Thus, NADPH was then used as the reducer. And we found that a Trp_779_ to Arg_783_ substituted OsNR2 (OsNR2-1112) exhibited significantly increased NR activity (Fig. 2c, d). Therefore, the Arg_783_ to Trp_779_ substitution in the NAD(P) binding domain of Nipponbare OsNR2 (Fig. 2a) reduces specific NR activity, conferring increased ClO_3_^-^ resistance, and indicating that Nipponbare *OsNR2* is a reduced function variant allele.

### *OsNR2* allelic divergence was driven by selection

**Fig. 3:**
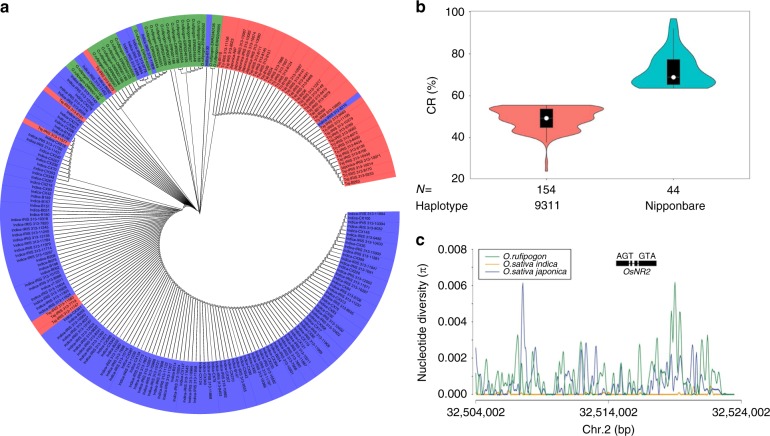
Phylogenetic and functional analysis of *OsNR2* haplotypes. **a** Phylogenetic tree of *OsNR2* CDS from 222 accessions. Blue: *O. sativa indica*; red: *O. sativa japonica*; green: *O. rufipogon*; Tej: *Temperate japonica*; Trj: *Tropical japonica*. **b** Classification of 199 cultivated rice varieties according to haplotype (9311 versus Nipponbare) and extent of conferred ClO_3_^-^ resistance (CR%). Error bars represent 95% confidence intervals. **c** DNA sequence diversity of the genomic region surrounding *OsNR2* in three groups. The green, orange and blue lines indicate site nucleotide diversity (π) for *O. rufipogon*, *O. sativa indica* and *O. sativa japonica* accessions, respectively. The position of *OsNR2* is as indicated (thick lines represent exons and thin lines introns). The source data underlying b are provided as a Source Data file

Phylogenetic analyses of *O.sativa indica*, *O.sativa japonica* and *O. rufipogon* accessions next determined the extent to which the 9311 and Nipponbare *OsNR2* alleles are representative of overall *indica* versus *japonica OsNR2* divergence (Fig. 3a) by F_st_ value (Supplementary Table 4). Further analysis of cultivated varieties identified two distinct *OsNR2* haplotypes defined by the three nonsynonymous SNPs distinguishing the 9311 and Nipponbare *OsNR2* alleles (Fig. 2a; Supplementary Fig. 3). Almost all *indica* varieties (147 of 148) carry the 9311 haplotype (conferring ClO_3_^−^ sensitivity), while most *japonica* varieties (43 of 51, including both temperate and tropical varieties) carry the Nipponbare haplotype (conferring ClO_3_^−^ resistance) (Fig. 3b). Thus, the 9311 versus Nipponbare *OsNR2* alleleic difference is largely reflective of overall *indica* versus *japonica OsNR2* allelic divergence, explaining why *indica* and *japonica* cultivars can be reliably distinguished in ClO_3_^−^ resistance assays.

Nucleotide diversity (π) analysis of *OsNR2* and neighboring regions (10 kb) showed reduction of 99.0% (*indica*) or 96.0% (*japonica*) of *O. rufipogon OsNR2* diversity, indicating that directional selection^23,24^ has driven the evolutionary divergence of the *indica* and *japonica OsNR2* alleles (Fig. 3c). Correspondingly, a neutrality test^25^ detected significant selection signatures for both alleles (Tajima’s D -1.96 for *indica* versus −2.11 for *japonica*; *P* < 0.05), the evidence of selective sweeps. Total of 100 genes were randomly selected from the sequenced *japonica* and *indica* genomes and average nucleotide diversity was calculated. The *OsNR2* nucleotide diversity was 9.06-fold and 3.75-fold lower than that of 100 randomly chosen gene fragments in the *indica* (π = 0.00280) and *japonica* (π = 0.00282) sample, respectively (Supplementary Data 1), suggesting that the lower nucleotide diversity observed in *OsNR2* loci of *indica* and *japonica* resulted from strong selection of it occurred during rice domestication. Furthermore, most *japonica* varieties (42 of 50) exhibited a ka/ks > 1, indicating that positive selection drove the divergence of the *japonica OsNR2* allele (Supplementary Fig. 6), despite its relatively reduced conferred NR function.

### The 9311 *OsNR2* allele promotes grain yield

**Fig. 4:**
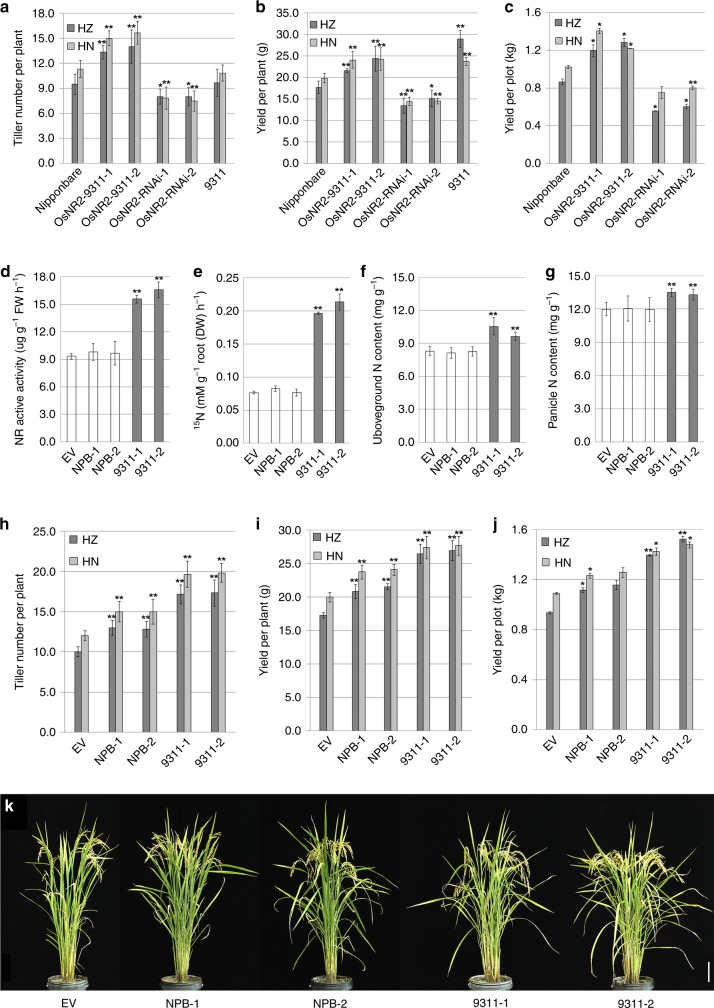
Expression of the 9311 *OsNR2* allele boosts Nipponbare N assimilation, tiller number, and grain yield. **a** effective tiller number, **b** yield per plant and **c** yield per plot of Nipponbare, Nipponbare harboring constructs for expression of the 9311 *OsNR2* allele driven by the 9311 *OsNR2* promoter (*OsNR2*-9311-1 and *OsNR2*-9311-2) or of *OsNR2* RNAi (*OsNR2-*RNAi-1 and *OsNR2-*RNAi-2). **d** NR active activity of Nipponbare (empty-vector control; EV), transgenic derivatives expressing the 9311 *OsNR2* allele (9311-1 and 9311-2), and transgenic derivatives expressing the Nipponbare *OsNR2* allele (NPB-1 and NPB-2), with expression of both alleles being driven by the CaMV 35S promoter. **e** ^15^NO_3_^-^ uptake of plants exposed to 1.25 mM ^15^NO_3_, **f** N content of aboveground plant parts, **g** panicle N content, **h** effective tiller number, **i** yield per plant, **j** yield per plot and **k** appearance of plant genotypes (as in (**d**)). Plants were cultivated in field conditions with NO_3_^−^ fertilizer (14 kg per acre) as major N source **f**–**i**. Values are mean ± s.d. (*n* = 6 for **a**-**b**, **f**–**i**, *n* = 3 for **d**, **e** and *n* = 2 for **c**, **j**). Error bar represents s.d. * and ** respectively indicate least significant differences at the 0.05 and 0.01 probability levels, compared with Nipponbare or EV. HZ: Hangzhou (harvested on September 20th, 2018); HN: Hainan (harvested on April 20th, 2019). Source data are provided as a Source Data file

*pOsNR2-GUS* fusion construct and qRT-PCR experiments indicated *OsNR2* was expressed in shoot and root vasculature, young root elongation zones, flowers and mostly in leaf (Supplementary Fig. 7). We next determined the effects of expression of the 9311 *OsNR2* allele on agronomic traits. First, we found that effective tiller number is respectively increased or decreased in Nipponbare plants expressing 9311 *OsNR2* (expression driven by the 9311 *OsNR2* promoter) or having reduced *OsNR2* mRNA abundance in Hangzhou (HZ) and Hainan (HN) (Fig. 4a, Supplementary Fig. 8). Correspondingly, the transcript abundances of *OsTB1*^26^, a gene controlling tiller bud formation and elongation, was reduced by expression of 9311 *OsNR2*, but increased by reduction in *OsNR2* mRNA abundance (Supplementary Fig. 9). Second, with effective tiller number being an important contributor to yield, we found that yield per plant and yield per plot in both HZ and HN were respectively increased or decreased in Nipponbare plants expressing 9311 *OsNR2* or having reduced *OsNR2* mRNA abundance (Fig. 4b, c). We next found that transgenic expression of the 9311 *OsNR2* allele (driven by the CaMV 35S promoter) increased the maximal and active NR activity, ^15^NO_3_^−^ uptake activity, and aboveground and panicle nitrogen contents of Nipponbare plants (9311-1 and 9311-2; Fig. 4d–g; Supplementary Fig. 2d). Transgenic expression of the 9311 *OsNR2* allele also conferred increased effective tiller number and grain yield on Nipponbare grown with NO_3_^−^ as the major N source in both HZ and HN, while Nipponbare effective tiller number and yield were also increased by transgenic expression of the Nipponbare *OsNR2* allele, these effects were not as great as seen with the 9311 allele (Fig. 4h–k, Supplementary Fig. 10a).

### Feed-forward interaction of *OsNR2* with *OsNRT1.1B*

**Fig. 5:**
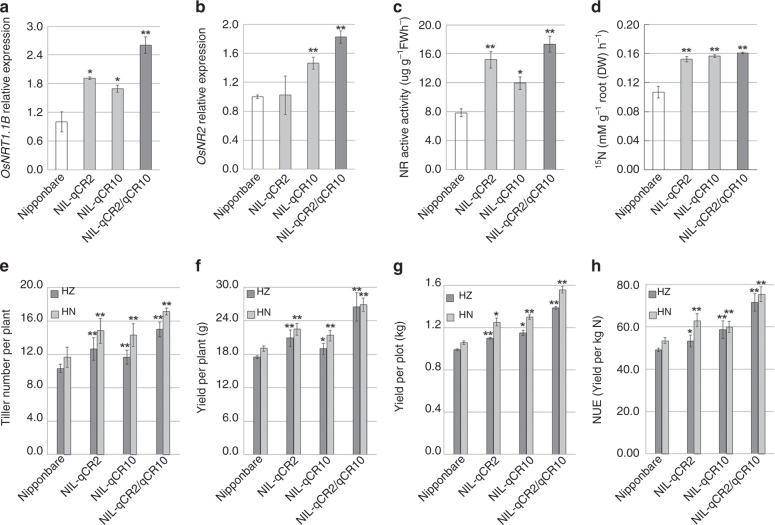
*OsNR2* and *OsNRT1.1B* interact to promote rice yield and NUE. **a** root *OsNRT1.1B* mRNA abundance, **b** leaf *OsNR2* mRNA abundance, **c** leaf NR active activity, **d** ^15^NO_3_^−^ uptake activity of roots exposed to 1.25 mM ^15^NO_3_^−^, **e** effective tiller number, **f** yield per plant, **g** yield per plot and **h** NUE of Nipponbare, NIL-*qCR2*, NIL-*qCR10* and NIL-*qCR2/qCR10*. Values are mean ± s.d. (*n* = 3 for **a**–**d**, *n* = 6 for **e**, **f**, **h** and *n* = 2 for **g**). Error bar represents s.d. Plants cultivated in field conditions with NO_3_^−^ fertilizer (14 kg per acre) as major N source **e**–**h**. * and ** respectively indicate least significant differences at the 0.05 and 0.01 probability levels, compared with Nipponbare. HZ: Hangzhou (harvested on September 20th, 2018); HN: Hainan (harvested on April 20th, 2019). Source data are provided as a Source Data file

Because *qCR2* (*OsNR2*) and *qCR10* (*OsNRT1.1B*^20^) have strong individual effects on ClO_3_^−^ resistance, we next investigated their inter-relationships and found that the 9311 *OsNR2* allele increases *OsNRT1.1B* mRNA abundance in both NIL-*qCR2* (Fig. 5a) and transgenics (Supplementary Fig. 10b). These observations suggest that the increased NO_3_^−^ uptake conferred by 9311 *OsNR2* (Figs. 1f and  4e) may be due to increased OsNRT1.1B activity. Reciprocally, the *indica OsNRT1.1B* allele (in NIL-*qCR10*) confers increased *OsNR2* mRNA abundance and enhanced NR activity (Fig. 5b, c). These observations imply a feed-forward amplifying relationship, with *OsNR2* function promoting *OsNRT1.1B* function, and *vice versa*. Indeed, a double Nipponbare NIL carrying both of the *indica OsNRT1.1B* and *OsNR2* alleles (NIL-*qCR2/qCR10*) displayed increased *OsNRT1.1B* and *OsNR2* mRNA abundances and NR activity above those of either single NIL (NIL-*qCR2* or NIL-*qCR10*) (Fig. 5a-c), resulting in NIL-*qCR2/qCR10* having the highest NO_3_^−^ uptake, aboveground N content, panicle N content, effective tiller number and yield per plant in both HZ and HN (Supplementary Fig. 11; Fig. 5e-f). The yield-enhancing increased effective tiller number of NIL-*qCR2/qCR10* is associated with corresponding decreases in *OsTB1* (Supplementary Fig. 12). Further analysis showed that the root ^15^NO_3_^−^ uptake activity and *OsNRT1.1B* mRNA abundance of all tested *indica* varieties is significantly higher than that of *japonica* varieties (Supplementary Fig. 13; Supplementary Table 5), further strengthening the conclusion that there is a feed-forward relationship between *OsNR2* and *OsNRT1.1B*. Finally, urea-fertilized (180 kg N ha^−1^) paddy field experiments again revealed NIL-*qCR2/qCR10* to have the highest yield per plot and NUE in HZ (cultivated from May to September, 2018) and HN (cultivated from December, 2018 to April, 2019) versus Nipponbare, NIL-*qCR2* or NIL-*qCR10* (Fig. 5g, h).

## Discussion

We have shown that allelic variation at *OsNR2* underlies the ClO_3_^−^ resistance QTL *qCR2*, and that *OsNR2* encodes a NAD(P)H-bispecific NR enzyme. Whilst NADH-specific NRs are relatively well understood^27^, little is known about the function of NAD(P)H-bispecific NRs in rice. Nevertheless, we have here shown that the OsNR2 (here NADPH mainly specific) is actually a major contributor to rice ClO_3_^−^ resistance, grain yield and NUE. Specifically, we have shown that the Arg_783_ residue characteristic of the NAD(P) binding domain of 9311 OsNR2 (but replaced by Trp_779_ in Nipponbare OsNR2) is responsible for the greater ClO_3_^-^ sensitivity and active NR activity conferred by 9311 *OsNR2* (versus that conferred by Nipponbare *OsNR2*) (Fig. 2).

In our study, haplotype analysis indicated that the 9311 and Nipponbare *OsNR2* alleles are largely representative of overall differences between *indica* and *japonica OsNR2* alleles. Phylogenetic analysis also indicated grouping of *indica OsNR2s* together with those from *O.rufipogon* accessions, suggesting greater divergence of *japonica OsNR2* from the presumed ancestral wild rice *OsNR2* (Fig. 3a). Furthermore, our ka/ks ratio analysis revealed evidence of positive selection^28^, with *OsNR2* exhibiting a ka/ks > 1 in 42 out of 50 *japonica* varieties (Supplementary Fig. 6). Thus, despite being a reduced function allele, *japonica OsNR2* was under positive selection during the differentiation of *indica* and *japonica*, perhaps during adaptation to distinct climatic, ecogeographic or cultural conditions. However, the correspondence between haplotype and varietal identity is not absolute, with several *japonica* varieties carrying the 9311 *OsNR2* haplotype and a single *indica* variety carrying the Nipponbare *OsNR2* haplotype. Nevertheless, ClO_3_^−^ resistance correlates strictly with *OsNR2* haplotype (Fig. 3b), further highlighting the key role of the Arg_783_ residue of 9311 OsNR2.

We have also identified a previously unknown regulatory relationship between OsNR2 nitrate reductase and OsNRT1.1B NO_3_^–^ uptake transporter functions in rice, showing that the 9311 *OsNR2* allele increases *OsNRT1.1B* function, while the 9311 *OsNRT1.1B* allele also increases OsNR2 function. While reciprocal regulation of NO_3_^−^ uptake and assimilation functions have previously been described in Arabidopsis^29–31^, there are potentially differences between Arabidopsis and rice in the specifics of their relationships. Further work will define in greater depth the nature of the feed-forward interaction between *OsNR2* and *OsNRT1.1B*.

Effective tiller number is an important contributory component of rice grain yield, and correlates positively with soil N availability^32^, with leaf and sheath N content influencing tiller emergence^33^. Although yield per plant of 9311 is significantly higher than Nipponbare both in HZ and HN, tiller number are similar between them (Fig. 4a, b). Therefore, increase in effective tiller number conferred by 9311 *OsNR2* allele was dependent on genetic background. Here, we have shown that the greater N-assimilative NR activity of *indica* OsNR2 (versus *japonica* OsNR2) confers enhanced tiller number, grain yield and NUE (Fig. 5c, e–h).

In conclusion, *indica* OsNR2 has greater specific activity than the diverged *japonica* OsNR2, and hence boosts the chlorate sensitivity ^15^NO_3_^−^ uptake and grain yield of *indica* varieties. NUE is further enhanced by the promotive feed-forward amplifying functional relationship between the *indica OsNRT1.1B* and *OsNR2* alleles, and this is particularly evident following introgression of both alleles into *japonica* varieties. Genetic variation at both *OsNR2* and *OsNRT1.1B* should therefore become major targets for breeders aiming to improve the world’s rice yield and the prospects for future global food security.

## Methods

### Development of plant materials and molecular markers

A total of 152 recombinant inbred lines (RILs) were derived from crossing the *japonica* variety Nipponbare with the *indica* variety 9311. These populations were subsequently developed in experimental fields at the China National Rice Research Institute in Hangzhou, Zhejiang Province, and in Lingshui, Hainan Province, China. To develop the NIL for the *qCR2* QTL (detected both in Hainan and Hangzhou), a RIL carrying the 9311 genotype in the *qCR2* region was selected for recurrent backcrossing with Nipponbare. SSR markers RM240 and RM250 (Supplementary Table 2) were used for marker-assisted selection (MAS) in each generation. As a result, a BC_4_F_1_ line, with a substantially Nipponbare genetic background but heterozyogous for DNA from 9311 and Nipponbare across the *qCR2* region was identified. Following self-pollination, derived BC_4_F_2_ and BC_4_F_3_ populations were obtained and used for the fine mapping of *qCR2*. Additionally, two NILs homozygous for 9311 DNA in the target QTL region between InDel markers IND2-1 and IND2-5, and SSR markers RM3123 and RM5666 (Supplementary Table 2) were respectively developed in the Nipponbare background and designated NIL-*qCR2* and NIL-*qCR10*. Subsequently, NIL-*qCR2/qCR10* was screened from the F_2_ of a cross between NIL-*qCR2* and NIL-*qCR10*. For the haplotype and phylogenetic analyses, 199 cultivated rice accessions were sampled, including 148 *O. sativa indica* and 51 *O. sativa japonica* accessions. All varieties were from the China National Rice Research Institute (Hangzhou, China) or the International Rice Research Institute (Philippines). Additional 23 *O.rufipogon* rice accessions were also sampled (Supplementary Table 6).

### Determination of ClO_3_^-^ resistance

ClO_3_^−^ resistance phenotypes were investigated at the seedling germination stage, using a method based on that described in Teng et al.^21^. Briefly, rice seeds were placed in a petri-dish on filter paper fully wetted with distilled water or with a 0.02% potassium chlorate solution, and then incubated at 28 °C, 14h-light/10h-dark period (RXZ-500D, Ningbo Jiangnan Instrument, China). The solution was changed every other day. In total 12 seedlings were measured in each sample, with ClO_3_^−^ resistance phenotypes being determined after 7 days (by measuring the length of each seedling). ClO_3_^−^ resistance was calculated as the following Equation **1**:

ClO_3_^−^ resistance (CR %) = (Average seedling length in ClO_3_^−^/Average seedling length in water) × 100%.(**1**).

### QTL mapping

A genetic map consisting of 134 InDel and SSR markers evenly distributed throughout the 12 rice chromosomes was constructed for QTL mapping. QTL analysis was performed with the MultiQTL package (www.multiqtl.com), using the maximum likelihood interval mapping approach for the RIL-selfing population. For major-effect QTLs, the LOD threshold was obtained based on a permutation test (1000 permutations) for each dataset. QTLs were named according to McCouch et al.^34^.

### DNA extraction and *OsNR2* sequencing

Genomic DNA was extracted from fresh rice leaves using the cetyl-trimethyl-ammonium bromide (CTAB) method^35^, with minor modifications. To obtain the *OsNR2* genomic DNA sequence, a 3026-bp region of the *OsNR2* gene from the start ATG to the TGA was amplified with LA Taq DNA polymerase in GC buffer I (Takara), using PCR sequencing primers NR3-NR8 (Supplementary Table 7). The PCR products were purified with a purification kit (Tiangen Company). All PCR products were sequenced directly on both strands with the primers.

### SNP calling, phylogenetic and genetic diversity analysis

We downloaded genome sequence data of 222 rice accessions from previous studies^36–38^. Rice reference genome (MSU v7.0) was downloaded from the Rice Genome Annotation Project (http://rice.plantbiology.msu.edu/pub/data/Eukaryotic_Projects/o_sativa/annotation_dbs/pseudomolecules/version_7.0/all.dir/). The genome sequence data of 147 *O.sativa indica* and 50 *O. sativa japonica* were obtained from NCBI (https://www.ncbi.nlm.nih.gov/biosample?Db=biosample&DbFrom=bioproject&Cmd=Link&LinkName=bioproject_biosample&LinkReadableName=BioSample&ordinalpos=1&IdsFromResult=262761). The sequence data of 23 *O. rufipogon* were downloaded from the NCBI BioProject PRJEB19404 (https://www.ncbi.nlm.nih.gov/bioproject/?term=PRJEB19404; ERR2240123, ERR2240125, ERR2240126, ERR2245548-ERR2245557), PRJDA39855 (https://www.ncbi.nlm.nih.gov/bioproject/?term=PRJDA39855; DRR000347, DRR000348) and PRJDB2009 (https://www.ncbi.nlm.nih.gov/bioproject/?term=PRJDB2009; DRR001183-DRR001190). Raw reads were trimmed with trimmomatic version 0.36 (http://www.usadellab.org/cms/uploads/supplementary/Trimmomatic/Trimmomatic-Src-0.36.zip) with parameters (ILLUMINACLIP:2:30:10 MINLEN:50 LEADING:20 TRAILING:20 SLIDINGWINDOW:5:20), and then aligned to the Nipponbare v7 reference genome (http://rice.plantbiology.msu.edu/) using BWA-MEM v0.7.10^39^. SNP calling and filtration were carried out with SAMtools version 1.6 (DP < 3 and a quality score < 30)^40^. SNP data from the *OsNR2* genomic sequences of 199 cultivated rice and 23 wild rice accessions are supplied in Supplementary Data 2-4. The SNP information from *LOC_Os02g53130* CDS was used to generate a maximum likehood phylogeny using MEGA version 5.0^41^ with bootstrap method and 1000 replications. Online tool EvolView version 2 (http://www.evolgenius.info/evolview/)^42^ was used to display the phylogenetic tree and for annotation. Calculation of population differentiation extent was estimated by R/Hierfstat package v0.04-22 using the F_st_ statistic^43^, with a 100-kb sliding window with 10-kb steps. Sequence diversity (π) and Tajima’s D were computed with DnaSP v6^44^ for each *O. sativa japonica*, *O. sativa indica* and *O. rufipogon* group^45^. Sequences were aligned by MAFFT (default parameters) and ka/ks value was computed using the Ka/Ks calculator 2.0^28^. Nucleotide diversity (π) of *OsNR2* and 100 randomly selected gene fragments were calculated with vcftools v0.1.13 (https://sourceforge.net/projects/vcftools/files/)^46^ with parameter (–site-pi) for *indica* and *japonica* groups. Sequence alignments were performed using ClustalX 2.0^47^. A PCR marker CR2-ID for InDel_2194_ is listed in Supplementary Table 7.

### Vector construction and plant transformation

A 9.773-kb 9311 genomic DNA fragment containing the entire *OsNR2* gene, 5356 bp of upstream sequence, and 1378 bp of downstream sequence was amplified with a high-fidelity DNA polymerase. This fragment was inserted into binary vector pCAMBIA1300, thus generating the transformation plasmid pCAMBIA1300-*OsNR2*-9311 that was used for the complementation test (Supplementary Fig. 1a). For plant transformation, the binary plasmid was first introduced into *Agrobacterium tumefaciens* EHA105 by electroporation, followed by transformation of Nipponbare callus^48^. Hyg primers for hygromycin resistance were used in the screening of transgenic plants (Supplementary Table 7). For RNAi analysis, Nipponbare was transformed with pTCK303-*OsNR2*-RNAi (Supplementary Fig. 1b), which contains nucleotides 1–119 of the *OsNR2* CDS amplified using the NR2RNAi primer (Supplementary Table 7). In addition, a 1.541-kb promoter fragment from Nipponbare *OsNR2* was amplified by high fidelity DNA polymerase using NR2P primers (Supplementary Table 7) and then inserted upstream of the 5 end of the *uidA* gene (encoding β-glucuronidase (GUS)) in pCAMBIA1301 (Supplementary Fig. 5a). The resultant pCAMBIA1301-*OsNR2p*:GUS fusion vector was transformed into Nipponbare via *Agrobacterium tumefaciens*.

### Expression and purification of OsNR2-GST fusion protein

The 5 ml overnight cultures of *E.coli* harboring GST-fusion protein expression constructs were added to 500 ml 2XTY medium with 150 mg ml^−1^ of ampicillin. The cultures were then shaken at 37 °C until reaching an A_600_ of 0.6, at which point protein expression was induced with 0.1 mM of IPTG. The cells were then pelleted 2 h after induction by centrifugation at 2655 × *g*, 4 °C for 10 min. The supernatant was discarded and the cells re-suspended in 10 ml ice-cold PBS. Next, 200 μl of freshly prepared 10 mg ml^-1^ lyozyme solution, 4 μl 20 μg ml^−1^ DNase I, 100 μl 100 mM L^−1^ MgCl_2_ and 100 μl 0.1 mol L^–1^ PMSF were added, and the mixture was incubated on ice for 30 min. The mixture was then sonicated with 300 W, at 3 s intervals for 20 min, then centrifuged at 15,294 × *g* at 4 °C for 10 min to pellet the debris. Next, the supernatant was transferred to a GST-Sefinose^TM^ pre-packed gravity column (Cat No: C600911, Sangon Biotech, Shanghai, China)^49^. In total 5 ml binding/wash buffer was twice added, followed by 2 ml elution buffer, which released the GST-fusion protein, which was then used for western blot analysis and determination of enzyme activity.

### Western blot analysis and NR activity measurement

Proteins were separated by 10% SDS–PAGE and western blotting was performed with Anti GST-Tag Mouse Monoclonal Antibody (1000 × dilution, Cat No: CW0084M, CWBIOtech, Beijing, China)^50^. Calibration curves were constructed using BSA (0.5 mg ml^−1^) as standard in the range from 0 to 20 μl measuring absorbance at 595 nm. The purified NR protein was analyzed by applying standard Bradford assays at 595 nm. Nitrate Reductase (NR) activity was measured as described^51^. Briefly, the reaction mixture contained 0.4 mL GST-fusion protein, 1.2 ml 0.1 mmol L^−1^ potassium phosphate buffer (pH 7.5), 0.1 mmol L^−1^ KNO_3_, and 0.4 ml 0.25 mmol L^−1^ nicotinamide adenine dinucleotide phosphate (NADPH). Controls substituted 0.1 mmol L^−1^ potassium phosphate buffer (pH 7.5) for the NADPH. After 30 min incubation the NO_2_^−^ produced was colorimetrically measured at 540 nm following addition of 10 g kg^−1^ sulfanilamide in 2 mol L^−1^ HCl and 0.2 g kg^−1^ N-(1-naphthyl)-ethylene-diammonium dichloride (NED). NR activity was expressed as μg NO_2_^−^ mg^−1^ protein h^−1^.

### Measurement of leaf NR activity

For enzyme extraction, leaf samples were harvested between 1400 and 1430 hours and ground in liquid nitrogen, following which 5 ml extraction buffer was added (per 1 g fresh tissue). The extraction buffer contained 25 mmol L^−1^ potassium phosphate buffer (pH 8.8), 10 mmol L^−1^ cysteine, and 1 mmol L^−1^ EDTA. Following continuous grinding until thawing, the supernatant was centrifuged at 20,000 × *g* for 20 min at 4 °C. NR activity was measured as described in the above paragraph except that the 0.4 ml GST-fusion protein was replaced by 0.4 ml extraction aliquots. NR activity was expressed as μg NO_2_^−^ g^−1^ FW h^−1^.

### Gene expression analysis

A real-time polymerase chain reaction (qPCR) approach was used to measure gene expression levels. Total rice leaf RNA was isolated using a Trizol reagent (Invitrogen Inc.). In total 2 µg DNAase-treated total RNA was reverse-transcribed using Moloney murine leukemia virus (M-MLV) reverse transcriptase (Promega Madison, WI) and Oligo(dT)_18_ primers (Promega, Madison, WI). Reverse-transcribed products were diluted to one-twentieth, following which 1 µl of the cDNA template was amplified using qPCR primers. Real time PCR amplification mixtures (10 μl) contained 50 ng template cDNA, 2 × SYBR Green PCR Master Mix (Applied Biosystems), and 200 nM forward and reverse primers. Reactions were performed using an ABI PRISM_7900HT Sequence Detector (Applied Biosystems). The relative expression level for each transcript was obtained by comparison with the expression of the *Actin* gene. Primers for tested genes and *Actin* are listed in Supplementary Table 7.

### GUS assays

Histochemical GUS analysis was performed as described with minor modifications^52^. Briefly, transgenic plant samples were incubated in X-Gluc buffer overnight at 37 °C. The stained tissues were observed and photographed using a stereomicroscope with a camera (Nikon SMZ1000).

### N content assays

For field-based assays, plants were grown at the China National Rice Research Institute with NO_3_^−^ fertilizer (14 kg per acre) as major N source. Fertilizer was applied three times during growth: as basal fertilizer (1–2 days before transplanting), tillering fertilizer (7 days after transplanting) and heading-stage fertilizer. N content was determined with the Auto Kjeldahl Nitrogen Analysis System (UDK152, VELP, Italy) using the Kjeldahl method^53^.

### ^15^NO_3_^−^ uptake activity and ^15^N accumulation determination

Hydroponic growth conditions for rice seedlings were IRRI culture medium at pH 5.5 but with 1.25 mM KNO_3_ as the N supply^54^. Rice seedlings were first grown in this medium for 8 weeks in the greenhouse, and then subsequently deprived of N for 3 days. The plants were then rinsed in 0.1 mM CaSO_4_ for 1 min, then transferred to a solution containing 1.25 mM 40% atom ^15^NO_3_^−^ (Shanghai Research Institute of Chemical Industry) for 5 min for ^15^N influx and 3 days for ^15^N accumulation, respectively, finally rinsed again in 0.1 mM CaSO_4_ for 1 min. Roots were separated from the shoots immediately following the final transfer to CaSO_4_. Samples were heated at 105 °C for 30 min, and then dried at 75 °C for 3 days. Dry weights were recorded as biomass values. ^15^N content measurements were performed by gas isotope mass spectrometer (MAT-271). Uptake activity was calculated as the amount of ^15^N taken up per unit weight of roots per unit time.

### Analyses of agronomic traits

Important agronomic traits including effective tiller number per plant and grain yield per plant were measured in Hangzhou and Hainan on a single-plant basis, with 6 plants being measured randomly for each line. All filled grains from a single plant were collected and dried at 50 °C in an oven for measurement of grain yield per plant. Randomly picked filled grains were used for 1000-grain weight measurements. Total plot grains (randomized block design) were collected in Hangzhou (September 20th, 2018) and Hainan (April 20th, 2019), with two replicates, and treated as described above for measurement of actual yield. Paddy field experiments were fertilized with urea (180 kg N ha^−1^), applied at 60% as basal fertilizer and 40% once during top-dressing per growing season.

### Reporting summary

Further information on research design is available in the Nature Research Reporting Summary linked to this article.

## Supplementary information

Supplementary Information

Reporting Summary

Description of Additional Supplementary Files

Supplementary Data 1

Supplementary Data 2

Supplementary Data 3

Supplementary Data 4

Source Data

## Data Availability

Data supporting the findings of this work are available within the article and its Supplementary Information file. A reporting summary for this article is available as a Supplementary Information file. The datasets generated and analyzed during the current study are available from the corresponding author upon request. The source data underlying Figs. 1d–f, 2c, 2d, 3b, 4a–j and 5, as well as Supplementary Figs. 2, 5, 6, 7e, 9, 10, 11a, 11b, 12, and 13 are provided as a Source Data file.
