# Supplementary material for: The indica nitrate reductase gene OsNR2 allele enhances rice yield potential and nitrogen use efficiency
Source: Nat Commun. 2019 Nov 15;10:5207. doi: 10.1038/s41467-019-13110-8 (PMC6858341; doi:10.1038/s41467-019-13110-8)
Supplement: Supplementary file 1 — Supplementary Information [file 41467_2019_13110_MOESM1_ESM.pdf]

# The *indica* nitrate reductase gene *OsNR2* allele enhances rice yield potential and nitrogen use efficiency

Gao *et al.*

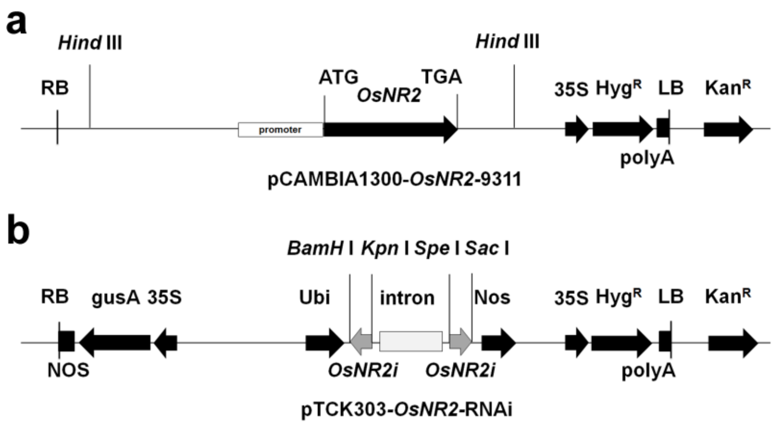


**Supplementary Fig. 1. Structures of constructs used for complementation test and RNAi analysis. a** Structure of pCAMBIA1300-*OsNR2*-9311 construct used for the complementation test. **b** Structure of pTCK303-*OsNR2*-RNAi construct used for the RNAi analysis.

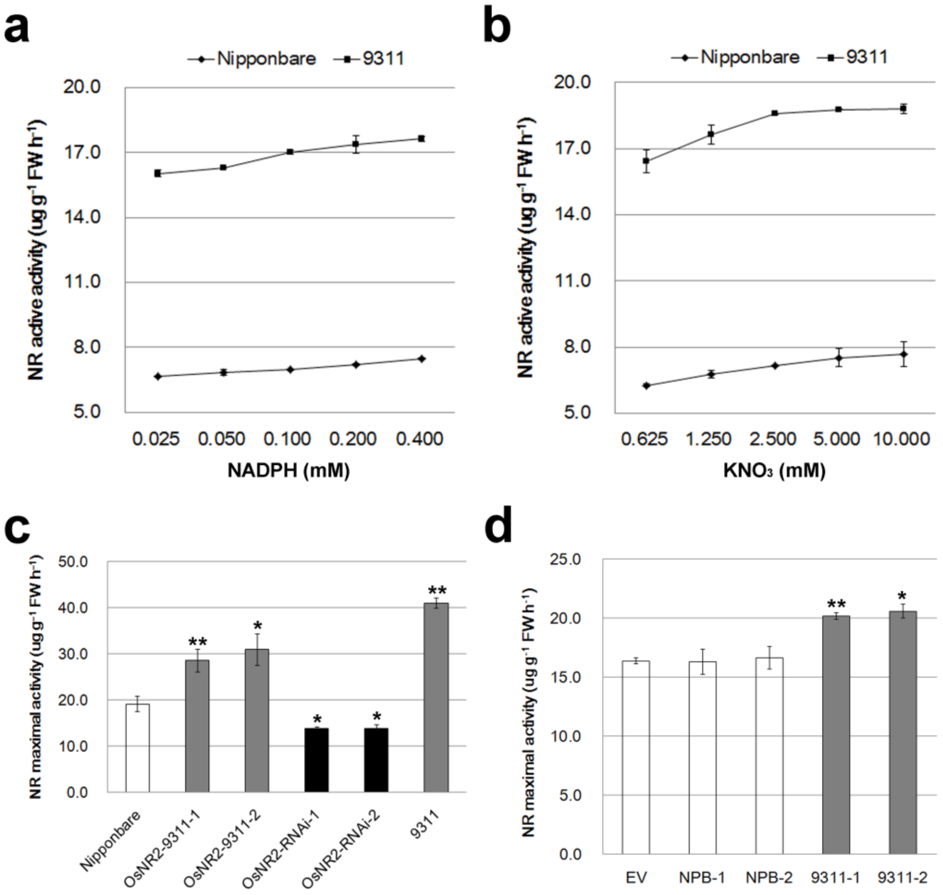


**Supplementary Fig. 2. NADPH and $NO_3^-$ concentration variation affects NR active activity and *OsNR2* allelic variation affects NR maximal activity. a** NR active activity sensitivity at varying NADPH concentration in Nipponbare and 9311. **b** NR active activity sensitivity at varying $NO_3^-$concentration in Nipponbare and 9311. **c** NR maximal activity in Nipponbare, 9311, Nipponbare lines expressing the 9311 (*indica*) allele (OsNR2-9311-1; OsNR2-9311-2), Nipponbare lines expressing an *OsNR2* RNAi construct (OsNR2-RNAi-1; OsNR2-RNAi-2). **d** NR maximal activity in Nipponbare (empty-vector control; EV), transgenic derivatives expressing the Nipponbare *OsNR2* allele (NPB-1 and NPB-2), transgenic derivatives expressing the 9311 *OsNR2* allele (9311-1 and 9311-2), expression of both alleles driven by the CaMV 35S promoter. Values are mean ± s.d. (n=3). Error bar represents s.d.. * and ** respectively indicate least significant differences at the 0.05 and 0.01 probability levels, compared with Nipponbare. Source data are provided as a Source Data file.

```
OsNR2-Nipponbare ATGGCGGCCTCGGTGGAGTACAAGCTGGCTCCGCACCCGTGGGCGAGCAATGCTCCGTCGAGCAACCTCGACTTGTTCCC   80
OsNR2-9311       ATGGCGGCCTCGGTGGAGTACAAGCTGGCTCCGCACCCGTGGGCGAGCAATGCTCCGTCGAGCAACCTCGACTTGTTCCC   80
OsNR2-Nipponbare GTCCGGCGGCGGCAAGCGCCGTTCGGGCTCCGAGACCGACTCCGACGACGAGGACAGCATACCACCGGACTGGAGGTCGC  160
OsNR2-9311       GTCCGGCGGCGGCAAGCGCCGTTCGGGCTCCGAGACCGACTCCGACGACGAGGACAGCATACCACCGGACTGGAGGTCGC  160
OsNR2-Nipponbare TGTACCACCCGCGGCTGGAGGTGGCGGAGCCGGCCGTCAAGGACCCCCGCGACGAGGCCACCTCCGACGCGTGGGTGCGT  240
OsNR2-9311       TGTACCACCCGCGGCTGGAGGTGGCGGAGCCGGCCGTCAAGGACCCCCGCGACGAGGCCACCTCCGACGCGTGGGTGCGT  240
OsNR2-Nipponbare CGCCACCCGGCGCTCGTCCGGCTCACCGGCAAGCACCCGTTCAACTCCGAGCCGCCGCTGCCGCGCCTCATGTCGCACGG  320
OsNR2-9311       CGCCACCCGGCGCTCGTCCGGCTCACCGGCAAGCACCCGTTCAACTCCGAGCCGCCGCTGCCGCGCCTCATGTCGCACGG  320
OsNR2-Nipponbare CTTCATCACGCCGGCGCCGCTCCACTACGTGCGCAACCACGGCGCCGTGCCCAAGGCGGACTGGTCGACGTGGGCCGTGG  400
OsNR2-9311       CTTCATCACGCCGGCGCCGCTCCACTACGTGCGCAACCACGGCGCCGTGCCCAAGGCGGACTGGTCGACGTGGGCCGTGG  400
OsNR2-Nipponbare AGGTGACCGGGCTCGTCAAGCGCCCCGCGAGGCTCAACATGGAGCAGCTCGTGACCGGGTTCGAGGCCGTGGAGCTCCCC  480
OsNR2-9311       AGGTGACCGGGCTCGTCAAGCGCCCCGCGAGGCTCACCATGGAGCAGCTCGTGACCGGGTTCGAGGCCGTGGAGCTCCCC  480
OsNR2-Nipponbare GTCACGCTGGTGTGCGCGGGCAACCGGCGCAAGGAGCAGAACATGGTGCGCCAGACCGTGGGCTTCAACTGGGGCCCCGG  560
OsNR2-9311       GTCACGCTGGTGTGCGCGGGCAACCGGCGCAAGGAGCAGAACATGGTGCGCCAGACCGTGGGCTTCAACTGGGGCCCCGG  560
OsNR2-Nipponbare CGCCATCTCCACCTCCGTGTGGCGCGGCGTGCGGCTGCGCGACGTGCTGCGGTGGTGCGGTGTCATGGGCGCCTCCGCCG  640
OsNR2-9311       CGCCATCTCCACCTCCGTGTGGCGCGGCGTGCGGCTGCGCGACGTGCTGCGGTGGTGCGGCGTCATGGGCGCCTCCGCCG  640
OsNR2-Nipponbare GCGCGGCCAACGTGTGCTTCGAGGGCGCCGAGGACCTCCCAGGCGGCGGCGGGTGCAAGTACGGCACCAGCCTGCGCCGC  720
OsNR2-9311       GCGCGGCCAACGTGTGCTTCGAGGGCGCCGAGGACCTCCCAGGCGGCGGCGGGTGCAAGTACGGCACCAGCCTGCGCCGC  720
OsNR2-Nipponbare GAGGTGGCCATGGACCCCGCCCACGACGTCATCCTCGCCTACATGCAGAACGGCGAGCCGCTCACGCCCGACCACGGCTT  800
OsNR2-9311       GAGGTGGCCATGGACCCCGCCCGCGACGTCATCCTCGCCTACATGCAGAACGGCGAGCCGCTCACGCCCGACCACGGCTT  800
OsNR2-Nipponbare CCCCGTCCGGGTCATCGTCCCGGGCTTCATCGGCGGCCGCATGGTGAAATGGCTCAAGCGCATCATCGTCGCGTCCAGCG  880
OsNR2-9311       CCCCGTCCGGGTCATCGTCCCGGGCTTCATCGGCGGCCGCATGGTGAAATGGCTCAAGCGCATCATCGTCGCGTCCAGCG  880
OsNR2-Nipponbare AGTCGGAGAGCTACTACCATTACCGCGACAACCGCGTCCTCCCGTCTCACGTCGACGCCGAGCTCGCCAATGCCGAAGgt  960
OsNR2-9311       AGTCGGAGAGCTACTACCATTACCGCGACAACCGCGTCCTCCCGTCTCACGTCGACGCCGAGCTCGCCAATGCCGAAGgt  960
OsNR2-Nipponbare aattaaccaaccatatcttcctcgaacgcactgcacgagacacacattgttgcatctttgtgatcttaacttgtgtgttt 1040
OsNR2-9311       aattaaccaaccatatcttcctcgaacgcactgcacgagacacacattgttgcatctttgtgatcttaacttgtgtgttt 1040
OsNR2-Nipponbare tttttttggattatgtgcagCTTGGTGGTACAAGCCGGAGTACATGATAAACGAGCTGAACATAAACTCGGTGATCACCA 1120
OsNR2-9311       tttttttggattatgtgcagCTTGGTGGTACAAGCCGGAGTACATGATAAACGAGCTGAACATAAACTCGGTGATCACCA 1120
OsNR2-Nipponbare CGCCGGGACACGATGAGGTGCTCCCCATCAATGCGCTGACGACGCAGCGGCCGTATACGATGAAGGGATACGCCTACTCC 1200
OsNR2-9311       CGCCGGGACACGATGAGGTGCTCCCCATCAATGCGCTGACGACGCAGCGGCCGTATACGATGAAGGGATACGCCTACTCC 1200
OsNR2-Nipponbare Ggtaagccccaagcccatctcatctatcatgttgtttgtttttgggcttcggcctgctcgttctaaaagcccaccatgat 1280
OsNR2-9311       Ggtaagccccaagcccatctcatctatcatgttgtttgtttttgggcttcggcctgctcgttctaaaagcccaccatgat 1280
OsNR2-Nipponbare cactttatgcttttactgggccgttataatatcagtatctttgtactgatggtgaatgggtgattaacggtgatgatttt 1360
OsNR2-9311       cactttatgcttttactgggccgttataatatcagtatctttgtactgatggtgaatgggtgattaacggtgatgatttt 1360
OsNR2-Nipponbare ctgttgccagGCGGTGGCCGGAAAGTTACAAGGGTAGAGGTGACCCTGGACGGCGGCGAGACGTGGCAGGTGTGCAACCT 1440
OsNR2-9311       ctgttgccagGCGGTGGCCGGAAAGTTACAAGGGTGGAGGTGACCCTGGACGGCGGCGAGACGTGGCAGGTGTGCAACCT 1440
OsNR2-Nipponbare TGACCACCCGGAGAGGCCGACCAAGTACGGAAAGTACTGGTGCTGGTGCTTCTGGTCCGTCGATGTCGAGGTGCTCGAGC 1520
OsNR2-9311       TGACCACCCGGAGAGGCCGACCAAGTACGGAAAGTACTGGTGCTGGTGCTTCTGGTCCGTCGATGTCGAGGTGCTCGAGC 1520
OsNR2-Nipponbare TGCTCGCCGCCAAGGAGATCGCCGTCCGCGCCTGGGACGAGTCCCTCAACACCCAGCCGGAGAAGCTCATTTGGAATCTC 1600
OsNR2-9311       TGCTCGCCGCCAAGGAGATCGCCGTCCGCGCCTGGGACGAGTCCCTCAACACCCAGCCGGAGAAGCTCATTTGGAATCTC 1600
OsNR2-Nipponbare ATGgtaagcactaagcaccagcaacggcaacgccatccgtttcaacgacgacgatgtgcttacgttttggttcgtgtgcg 1680
OsNR2-9311       ATGgtaagcactaagcaccagcaacggcaacgccatccgtttcaacgacgacgatgtgcttacgttttggttcgtgtgcg 1680
OsNR2-Nipponbare tgtgtgcagGGCATGATGAACAACTGCTGGTTCAGGGTGAAGACGAAGACGTGCAGGCCGCACAAGGGGGAGATCGGGCT 1760
OsNR2-9311       tgtgtgcagGGCATGATGAACAACTGCTGGTTCAGGGTGAAGACGAAGACGTGCAGGCCGCACAAGGGGGAGATCGGGCT 1760
OsNR2-Nipponbare GGTGTTCGAGCACCCGACGCAGCCGGGCAACCAGGCCGGCGGGTGGATGGCGAGGCAGAAGCACCTCGAGACGTCGGAGA 1840
OsNR2-9311       GGTGTTCGAGCACCCGACGCAGCCGGGCAACCAGGCCGGCGGGTGGATGGCGAGGCAGAAGCACCTCGAGACGTCGGAGA 1840
OsNR2-Nipponbare GCGCGGTGAGCACGCTGAAGCGCAGCACGTCCACGCCGTTCCTCAACACGGCCACCACGCAGTACACCATGTCCGAGGTG 1920
OsNR2-9311       GCGCGGTGAGCACGCTGAAGCGCAGCACGTCCACGCCGTTCCTCAACACGGCCACCACGCAGTACACCATGTCCGAGGTG 1920
OsNR2-Nipponbare CGCCGCCACACGACGCCGGAGTCCGCCTGGATCATCGTGCACGGCCATGTCTACGACTGCACGGGGTTCCTCAAGGACCA 2000
OsNR2-9311       CGCCGCCACACGACGCCGGAGTCCGCCTGGATCATCGTGCACGGCCATGTCTACGACTGCACCGGGTTCCTCAAGGACCA 2000
OsNR2-Nipponbare CCCCGGCGGCGCCGACAGCATCATGATCAACGCCGGCACCGACTGCACCGAGGAATTCGACGCCATCCACTCCGACAAGG 2080
OsNR2-9311       CCCCGGCGGCGCCGACAGCATCATGATCAATGCCGGCACCGACTGCACCGAGGAGTTCGACGCCATCCACTCCGACAAGG 2080
OsNR2-Nipponbare CCCGTGGCCTCCTCGAGATGTACCGCATCGGCGAGCTCATCGTCACCGGCAGCGACTACTCGCCGCAGAGCAGCAGTGCT 2160
OsNR2-9311       CCCGTGGCCTCCTCGAGATGTACCGCATCGGCGAGCTCATCGTCACCGGCAGCGACTACTCGCCGCAGAGCAGCAGTGCT 2160
OsNR2-Nipponbare GACCTCACGTCCATCGTTGAGAGCCCTACGGCA------------GCGGCGGCGCCCGCCGTGCCGGTGTCGACCGTCGC 2240
OsNR2-9311       GACCTCACGTCCATCGTTGAGAGCCCTACGGCAGCGGCGGCGGCGGCGGCGGCGCCCGCCGTGCCGGTGTCGACCGTCGC 2240
OsNR2-Nipponbare GCTGTCCAACCCGCGGGAGAAGGTGAAATGCCGGCTCATGGACAAGAAGAGCCTGTCCTACAACGTGCGCCTGTTCCGGT 2320
OsNR2-9311       GCTGTCCAACCCGCGGGAGAAGGTGAAATGCCGGCTCATGGACAAGAAGAGCCTGTCCTACAACGTGCGCCTGTTCCGGT 2320
OsNR2-Nipponbare TCGCGCTGCCGTCGCCGGACCAGAAGCTCGGGCTACCGGTCGGCAAGCACGTGTACGTGTGCGCGTCGATCGGCGGCAAG 2400
OsNR2-9311       TCGCGCTGCCGTCGCCGGACCAGAAGCTCGGGCTACCGGTCGGCAAGCACGTGTACGTGTGCGCGTCGATCGGCGGCAAG 2400
OsNR2-Nipponbare CTCTGCATGCGCGCGTACACGCCGACGAGCTCCGTCGACGAGGTCGGCTACATCGAGCTCCTGATCAAGATATACTTCAA 2480
OsNR2-9311       CTCTGCATGCGCGCGTACACGCCGACGAGCTCCGTCGACGAGGTCGGCTACATCGAGCTCCTGATCAAGATATACTTCAA 2480
OsNR2-Nipponbare GGGCGAGGACCCCAAGTTCCCCGACGGCGGGCTCATGTCGCAGTACCTGGACTACCTGCCGCTCGGCGCCACCATCGACA 2560
OsNR2-9311       GGGCGAGGACCCCAAGTTCCCCGACGGCGGGCTCATGTCGCAGTACCTGGACTACCTGCCGCTCGGCGCCACCATCGACA 2560
OsNR2-Nipponbare TCAAGGGCCCGATCGGGCACATCGAGTACGCCGGCCGCGGCGCCTTCACGGTGAACGGCGAGCGCCGGTTCGCGCGGCGG 2640
OsNR2-9311       TCAAGGGCCCGATCGGGCACATCGAGTACGCCGGCCGCGGCGCCTTCACGGTGAACGGCGAGCGCCGGTTCGCGCGGCGG 2640
OsNR2-Nipponbare CTCGCCATGGTGGCCGGCGGGACGGGGATCACGCCGGTGTACCAGGTGATCCAGGCCGTGCTCTGGGACCAGCCCGACGA 2720
OsNR2-9311       CTCGCCATGGTGGCCGGCGGGACGGGGATCACGCCGGTGTACCAGGTGATCCAGGCCGTGCTCAGGGACCAGCCCGACGA 2720
OsNR2-Nipponbare CGGAACGGAGATGCACGTGGTGTACGCGAACCGGACGGAGGACGACATGCTCCTCCGGGAGGAGATCGACCGGTGGGCGG 2800
OsNR2-9311       CGGAACGGAGATGCACGTGGTGTACGCGAACCGGACGGAGGACGACATGCTCCTCCGGGAGGAGATCGACCGGTGGGCGG 2800
OsNR2-Nipponbare CGGCGCACCCGGCGCGGCTCAAGGTGTGGTACGTGGTGAGCAAGGTGGCGCGGCCGGAGGACGGGTGGGAGTACGGCGTG 2880
OsNR2-9311       CGGCGCACCCGGCGCGGCTCAAGGTGTGGTACGTGGTGAGCAAGGTGGCGCGGCCGGAGGACGGGTGGGAGTACGGCGTG 2880
OsNR2-Nipponbare GGGAGGGTGGACGAGCGGACGCTCAGGGAGCACCTGCCGCCGGGCGACGGCGAGACGCTCGCGCTCGTGTGCGGGCCGCC 2960
OsNR2-9311       GGGAGGGTGGACGAGCGGACGCTCAGGGAGCACCTGCCGCCGGGCGACGGCGAGACGCTCGCGCTCGTGTGCGGGCCGCC 2960
OsNR2-Nipponbare GGCGATGGTCGAGTGCACGGTGCGGCCGGGCCTGGAGAAGATGGGCTATGACCTCGACAAGTCCTGCCTCGTCTTCTGA  3039
OsNR2-9311       GGCGATGGTCGAGTGCACGGTGCGGCCGGGCCTGGAGAAGATGGGCTATGACCTCGACAAGTCCTGCCTCGTCTTCTGA  3039
```

**Supplementary Fig. 3. DNA sequence alignment of protein-encoding and intron regions of the Nipponbare and 9311 *OsNR2* alleles.** Synonymous and nonsynonymous SNPs are respectively shown with grey and yellow backgrounds. Intron sequence is displayed in lowercase letters. Two distinct 9311 and Nipponbare *OsNR2* haplotypes are defined by the three nonsynonymous SNPs, $SNP_{437}$ (A/C), $SNP_{743}$ (A/G), $SNP_{2692/2704}$ (T/A) and by the $InDel_{2194}$ (D/I).

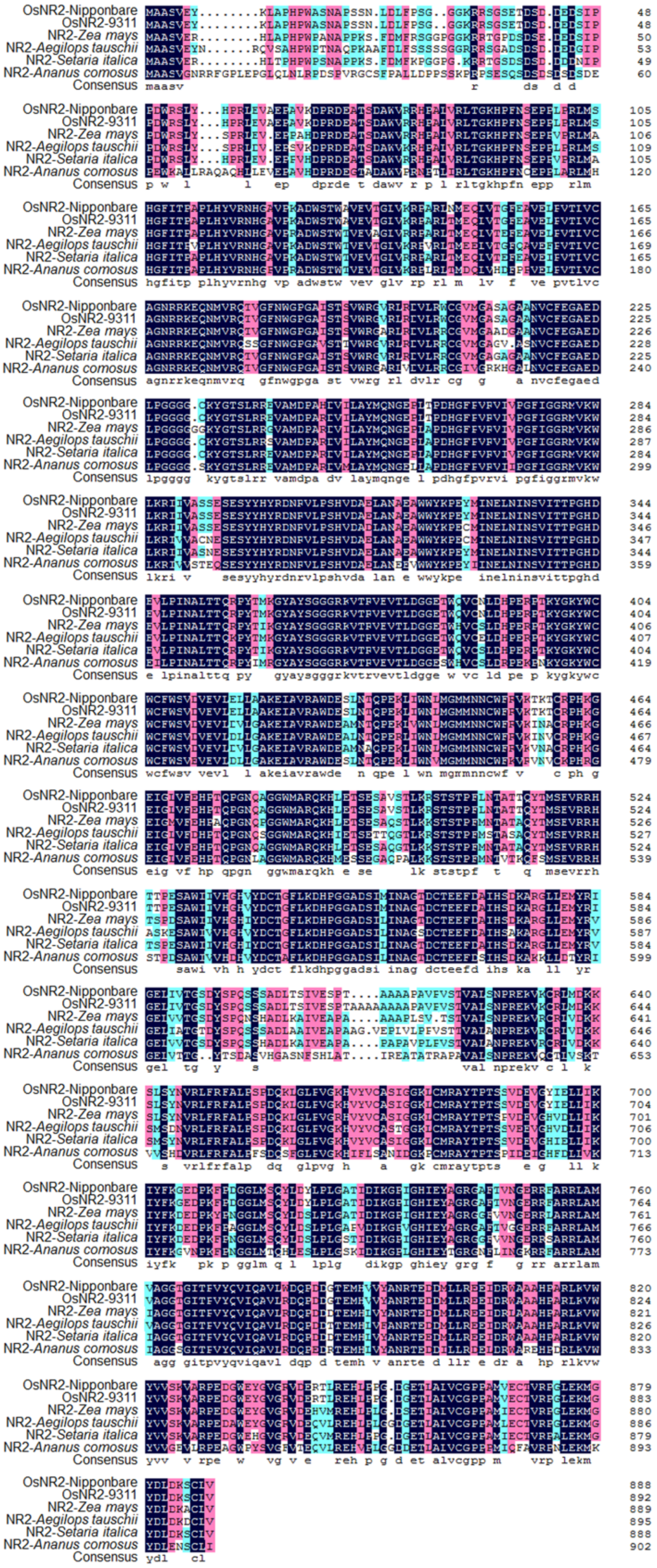

**Supplementary Fig. 4. Amino acid sequence alignment of orthologous to OsNR2 from four plant species with those of 9311 and Nipponbare OsNR2.** Four plant species include *Zea mays* (maize, NP_001169343), *Aegilops tauschii* (goat-grass, XP_020171964), *Setaria italica* (millet, XP_004954055), *Ananus comosus* (pineapple, XP_020091032).

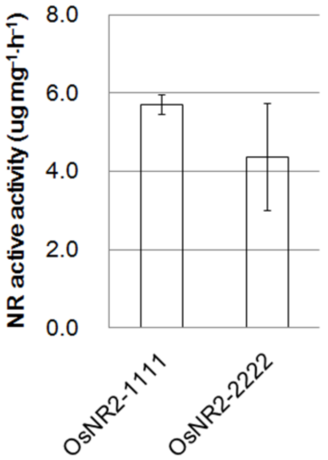


**Supplementary Fig. 5. NR active activity of purified extracts in *E.coli* expressing Nipponbare and 9311 OsNR2 proteins with NADH as a reducer.** OsNR2-1111 and OsNR2-2222 represent Nipponbare and 9311 OsNR2, respectively. Values are mean ± s.d. (n=3). Error bar represents s.d.. Source data are provided as a Source Data file.

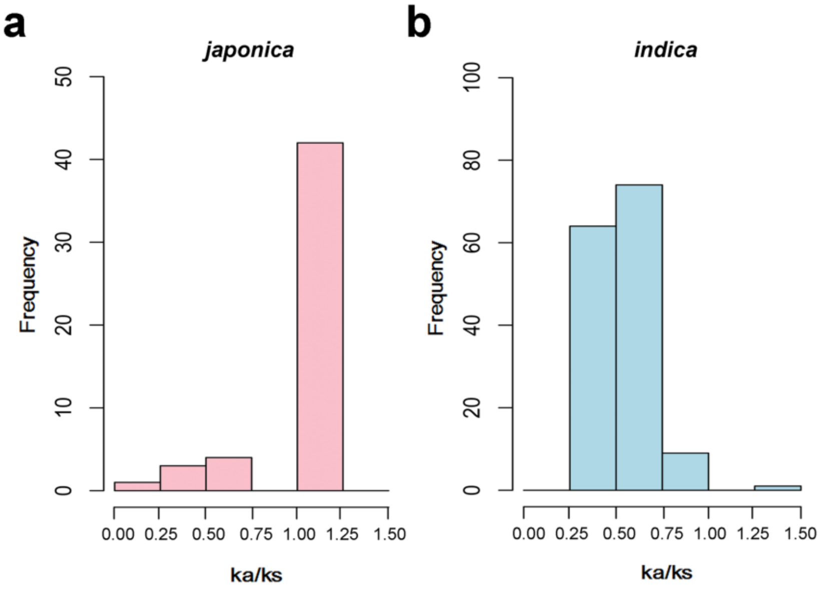


**Supplementary Fig. 6. *OsNR2* ka/ks frequency distribution in *japonica* and *indica* varieties. a** *OsNR2* ka/ks frequency distribution in *japonica*. **b** *OsNR2* ka/ks frequency distribution in *indica*. Source data are provided as a Source Data file.

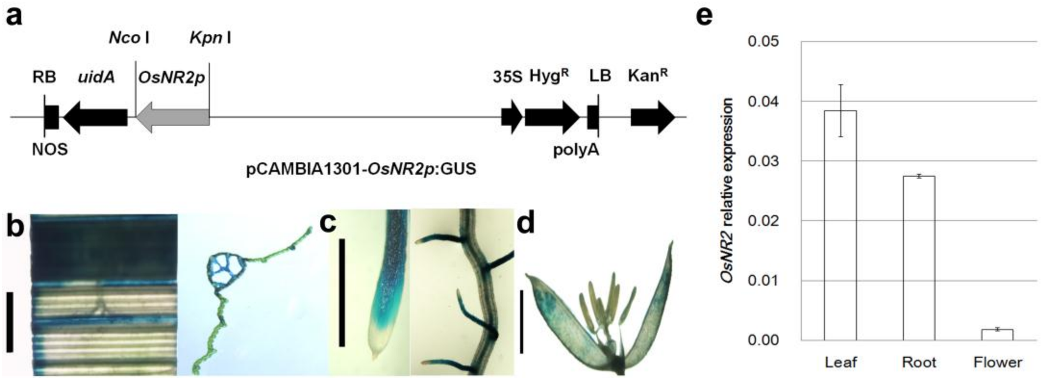


**Supplementary Fig. 7. Tissue expression pattern of *OsNR2*. a** Structure of pCAMBIA1300-*pOsNR2*:GUS construct. Localization of *pOsNR2:GUS* expression in rice (**b**) leaf longitudinal and horizontal sections, (**c**) longitudinal section of a root, lateral roots, and (**d**) flower. Scale bars = 1 cm. **e** *OsNR2* mRNA abundance in rice leaf, root and flower. Values are mean ± s.d. (n=3). Error bar represents s.d.. The source data underlying Supplementary Figure 7e are provided as a Source Data file.

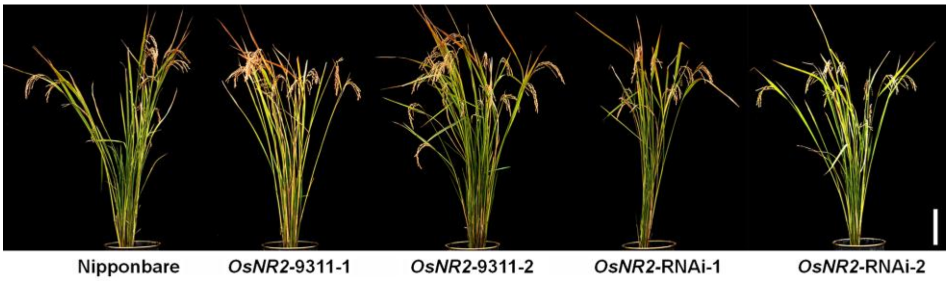


**Supplementary Fig. 8. Mature plant phenotypes and tiller morphologies of Nipponbare and transgenic lines.** *OsNR2*-9311-1 and *OsNR2*-9311-2 represent Nipponbare harboring constructs for expression of the 9311 *OsNR2* allele driven by the 9311 *OsNR2* promoter; *OsNR2*-RNAi-1 and *OsNR2*-RNAi-2 represent Nipponbare harboring constructs for *OsNR2* RNAi. Scale bar =10 cm.

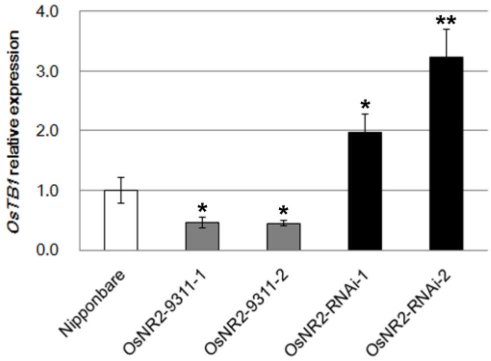


**Supplementary Fig. 9. Relative *OsTB1* mRNA abundances in Nipponbare and transgenic lines.** *OsNR2*-9311-1 and *OsNR2*-9311-2 represent Nipponbare harboring constructs for expression of the 9311 *OsNR2* allele driven by the 9311 *OsNR2* promoter; *OsNR2*-RNAi-1 and *OsNR2*-RNAi-2 represent Nipponbare harboring constructs for *OsNR2* RNAi. Values are mean ± s.d. (n=3). Error bar represents s.d.. * and ** indicate the least significant difference at 0.05 and 0.01 probability level compared with Nipponbare, respectively. Source data are provided as a Source Data file.

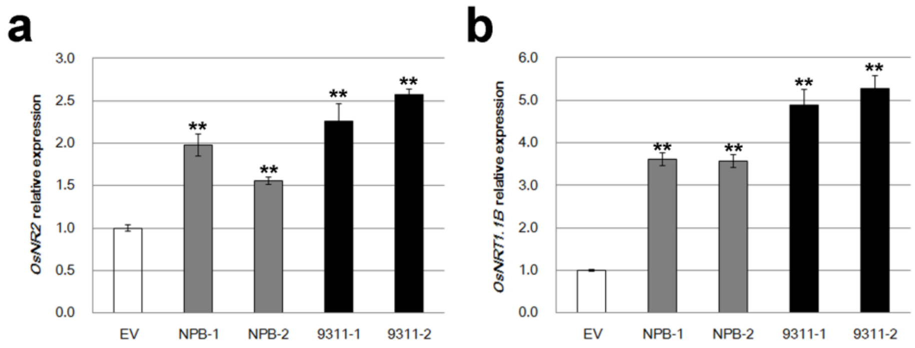


**Supplementary Fig. 10. Relative *OsNR2* and *OsNRT1.1B* mRNA abundances in Nipponbare transformed with empty vector and transgenic *OsNR2* derivatives.** Relative *OsNR2* mRNA abundances (**a**) and relative *OsNRT1.1B* mRNA abundances (**b**) in EV (Nipponbare transformed with empty vector), transgenic derivatives expressing the 9311 *OsNR2* allele (9311-1 and 9311-2), and transgenic derivatives expressing the Nipponbare *OsNR2* allele (NPB-1 and NPB-2), expression of both alleles driven by the CaMV 35S promoter. Values are mean ± s.d. (n=3). Error bar represents s.d.. ** indicate the least significant difference at 0.01 probability level compared with Nipponbare. Source data are provided as a Source Data file.

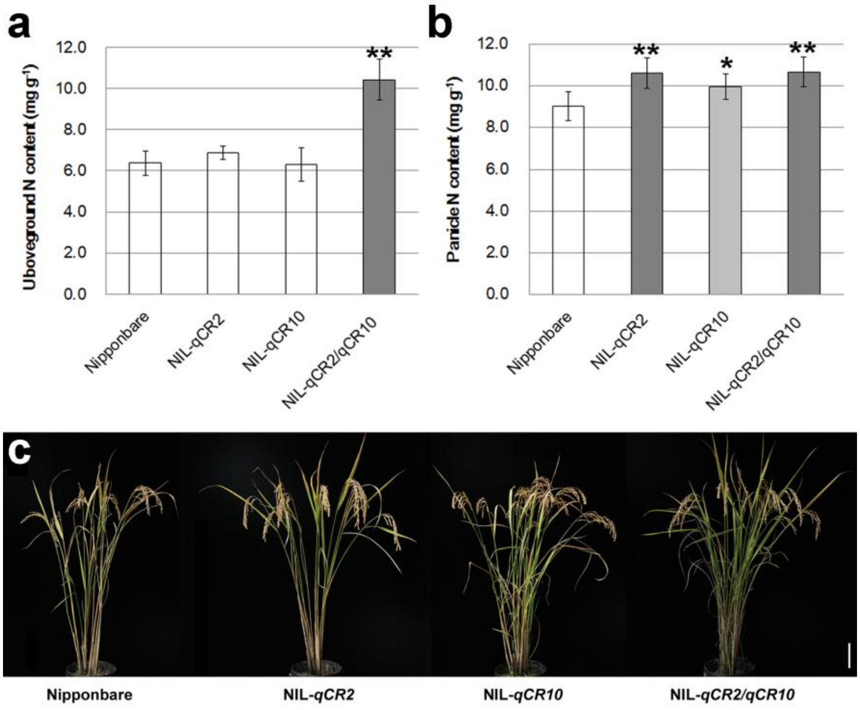


**Supplementary Fig. 11. N content of aboveground plant parts and panicles, and appearance of Nipponbare and NILs for *qCR2, qCR10* and both.** aboveground N content (**a**) and panicle N content (**b**) of Nipponbare, NIL-*qCR2*, NIL-*qCR10* and NIL-*qCR2/qCR10*. Values are mean ± s.d. (n=3). Error bar represents s.d.. * and ** indicate the least significant difference at 0.05 and 0.01 probability level compared with Nipponbare, respectively. **c** Mature plant phenotypes and tiller morphologies of Nipponbare, NIL-*qCR2*, NIL-*qCR10* and NIL-*qCR2/qCR10*. Scale bar =10 cm. Plants cultivated in field conditions with $NO_3^-$ fertilizer (14kg per acre) as major N source in HZ (harvested on September 20th, 2018). The source data underlying Supplementary Figure 11a and 11b are provided as a Source Data file.

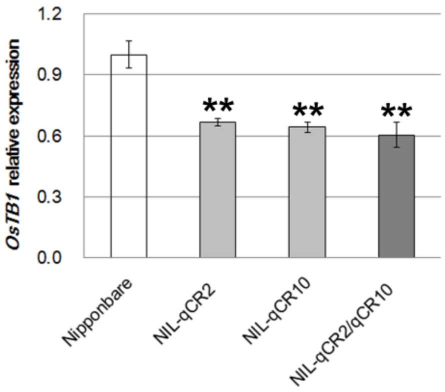


**Supplementary Fig. 12. Relative *OsTB1* mRNA abundances in Nipponbare, NIL-*qCR2*, NIL-*qCR10* and NIL-*qCR2/qCR10*.** Values are mean ± s.d. (n=3). Error bar represents s.d.. ** indicates the least significant difference at 0.01 probability level compared with Nipponbare. Source data are provided as a Source Data file.

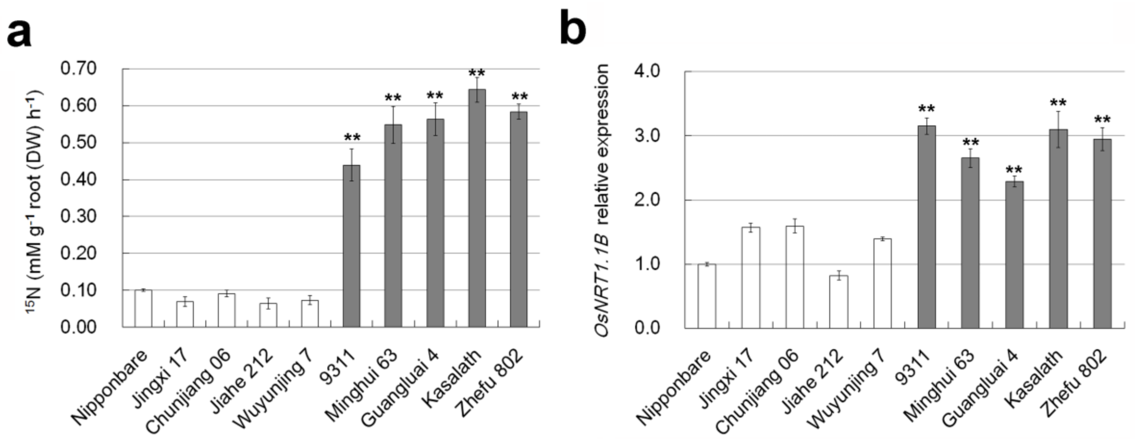


**Supplementary Fig. 13. Root $^{15}NO_3^-$ uptake and relative *OsNRT1.1B* mRNA abundances of five *japonica* and five *indica* varieties exposed to 1.25 mM $^{15}NO_3^-$.** Root $^{15}NO_3^-$ uptake (**a**) and relative *OsNRT1.1B* mRNA abundances (**b**) of Nipponbare (*japonica*), Jingxi 17 (*japonica*), Chunjiang 06 (*japonica*), Jiahe 212 (*japonica*), Wuyunjing 7 (*japonica*), and 9311 (*indica*), Minghui 63 (*indica*), Guangluai 4 (*indica*), Kasalath (*indica*), Zhefu 802 (*indica*). Values are mean ± s.d. (n=3). Error bar represents s.d.. ** indicates least significant difference at the 0.01 probability levels (versus Nipponbare). Source data are provided as a Source Data file.

**Supplementary Table 1. QTLs for chlorate resistance of seeds from Hangzhou (HZ) and Hainan (HN).**

| QTL | Site | Chromosome | LOD | P value | Genetic distance (cM) | PVE (%) | SE | Marker |
|---|---|---|---|---|---|---|---|---|
| *qCR1* | HZ | 1 | 3.03 | 0.01 | 171.42-195.83 | 9.5 | 14.5 | 1-6~1-21 |
| *qCR2* | HZ | 2 | 2.82 | 0.01 | 155.88-181.87 | 8.0 | -13.3 | 2-22~2-26 |
| | HN | 2 | 4.98 | 0.01 | 155.88-175.33 | 23.8 | -19.5 | 2-22~2-24 |
| *qCR7* | HZ | 7 | 2.99 | 0.01 | 89.17-115.24 | 8.3 | 13.5 | 7-7~7-15 |
| *qCR10* | HZ | 10 | 2.30 | 0.01 | 86.67-105.56 | 7.0 | -12.6 | 10-5~10-7 |
| | HN | 10 | 2.26 | 0.01 | 86.67-112.20 | 12.6 | -14.1 | 10-5~10-8 |

PVE: Phenotype variance explained. SE: Substitution effect.

**Supplementary Table 2. Molecular markers in *qCR2* region.**

| Primer Name | Forward Primer (5'- 3') | Reverse Primer (5'- 3') |
|---|---|---|
| RM250 | GGTTCAAACCAAGCTGATCA | GATGAAGGCCTTCCACGCAG |
| IND4853 | ATGCTGACTGTTCATGTGCT | TGTAAAATTTGGTACATCATGC |
| IND4068 | AACGAGCTCTAGCGTGGGAC | CATGCGATTCTTACCACGTAG |
| IND2-1 | AGGCCCTAAAATCAACAATG | GATAATTTTATCACGGCAACG |
| IND2-2 | ATATCGCAAGTTAGGCTGGT | AAACAAAAGCAGCAGTAGCTAC |
| IND2-3 | AAATTAAAAGTAGAGTTTAGAGTCCGT | TCAGGATGGCATCTGTTCTTG |
| IND2-5 | TTCGAGGATTGATTTGACTAG | CTTTTCCTGTATCTATCTTGG |
| IND4080 | GGTTAACATATGCACCGTGAG | TCCACTTCTTTTGCGAGTTAG |
| IND4159 | TCGTGTAGAAACCATGTCTTG | CATTCCCAATGCAATGACTA |
| RM240 | CCTTAATGGGTAGTGTGCAC | TGTAACCATTCCTTCCATCC |

**Supplementary Table 3. Km of NR active activity for NADPH and nitrate in Nipponbare and 9311.**

| | Nipponbare | 9311 |
|---|---|---|
| NADPH | 0.0029 | 0.0018 |
| nitrate | 0.1474 | 0.1034 |

**Supplementary Table 4. Comparison of $F_{st}$ value.**

| | *O.sativa indica* vs *O.Rufipogon* | *O.sativa japonica* vs *O.Rufipogon* | *O.sativa indica* vs *O.sativa japonica* |
|---|---|---|---|
| Top 5% of whole genome | 0.298 | 0.361 | 0.514 |
| *OsNR2* gene region | 0.341 | 0.556 | 0.645 |

**Supplementary Table 5. Haplotype of *OsNR2* in five *japonica* and five *indica* varieties.**

| **Accession** | **Group** | **$SNP_{437}$** | **$SNP_{743}$** | **$SNP_{2704/2692}$** | **$InDel_{2194}$** | **Haplotype** |
|---|---|---|---|---|---|---|
| Nipponbare | *Temperate japonica* | T | T | A | N | Nipponbare |
| Jingxi 17 | *Temperate japonica* | T | T | A | N | Nipponbare |
| Chunjiang 06 | *Temperate japonica* | T | T | A | N | Nipponbare |
| Jiahe 212 | *Temperate japonica* | T | T | A | N | Nipponbare |
| Wuyujing 7 | *Temperate japonica* | T | T | A | N | Nipponbare |
| 9311 | *Indica* | G | C | T | 9 | 9311 |
| Minghui 63 | *Indica* | G | C | T | 9 | 9311 |
| Guangluai 4 | *Indica* | G | C | T | 9 | 9311 |
| Kasalath | *Indica* | G | C | T | 9 | 9311 |
| Zhefu 802 | *Indica* | G | C | T | 9 | 9311 |

**Supplementary Table 6. *O.rufipogon* accessions used for *OsNR2* sequence analysis.**

| **Accession** | **SNP$_{437}$** | **SNP$_{743}$** | **SNP$_{2704/2692}$** |
|---|---|---|---|
| DRR000347 | G | C | T |
| DRR000348 | G | C | T |
| DRR001183 | G | C | T |
| DRR001184 | G | C | T |
| DRR001185 | G | C | T |
| DRR001186 | G | C | T |
| DRR001187 | G | C | T |
| DRR001188 | G | C | T |
| DRR001189 | G | C | T |
| DRR001190 | G | C | T |
| ERR2240123 | G | C | T |
| ERR2240125 | G | C | T |
| ERR2240126 | G | C | T |
| ERR2245548 | G | C | T |
| ERR2245549 | G | C | T |
| ERR2245550 | G | C | T |
| ERR2245551 | G | C | T |
| ERR2245552 | G | C | T |
| ERR2245553 | G | C | T |
| ERR2245554 | G | C | T |
| ERR2245555 | G | C | T |
| ERR2245556 | G | C | T |
| ERR2245557 | G | C | T |

**Supplementary Table 7. Primers for sequencing, vector construction and real time qPCR.**

| Primer Name | Forward Primer (5'- 3') | Reverse Primer (5'- 3') |
|---|---|---|
| NR3 | GTCCGGCTCACCGGCAAG | GGTTGTCGCGGTAATGGTAGTAGC |
| NR4 | TCATGTCGCAGTACCTGGACTAC | GTATATGTACAGTTTTTGCAGTGTCGTAC |
| NR6 | TCCCCGTCCGGGTCATCGTC | CACGTCTTCGTCTTCACCCTGAACC |
| NR7 | ATGGGAGGCTGCTACAGGGATG | ACGTCGACCAGTCCGCCTTG |
| NR8 | AATCTCATGGTAAGCACTAAGCAC | TACTCGATGTGCCCGATCG |
| CR2-ID | TGACCTCACGTCCATCGTTGAGAGC | GTTGGACAGCGCGACGGTCG |
| Hyg | GGAGCATATACGCCCGGAGT | GTTTATCGGCACTTTGCATCG |
| NR2RNAi | GGTACCACTAGTATGGCGGCCTCGGTG | GGATCCGAGCTCGGTCTCGGAGCCCG |
| NR2P | GGTACCTCGAAATTAAGAAAAATATGAGC | CCATGGTGTTGCCGATGGAAGGACACA |
| *OsNR2* | TGGGGAGGGTGGACGAGCGGAC | AGCGTCTCGCCGTCGCCCG |
| *OsNRT1.1B* | AGGCTCGACTACTTCTACTGGC | TGAAGAGGACGAGGTTGATGG |
| *OsTB1* | TGATCATGGCGTTCGCCAACG | AGCTCCCACTGATCCTGCTGATGC |
| *Actin* | TCCATCTTGGCATCTCTCAG | GTACCCTCATCAGGCATCTG |
