# Supplementary material for: The indica nitrate reductase gene OsNR2 allele enhances rice yield potential and nitrogen use efficiency
Source: Nat Commun. 2019 Nov 15;10:5207. doi: 10.1038/s41467-019-13110-8 (PMC6858341; doi:10.1038/s41467-019-13110-8)
Supplement: Supplementary file 3 — Description of Additional Supplementary Files [file 41467_2019_13110_MOESM3_ESM.doc]

**Description of Additional Supplementary Files**

File Name: Supplementary Data 1.
Description: Nucleotide diversity of OsNR2 and 100 genes randomly selected from the japonica and indica genomes.

File Name: Supplementary Data 2.
Description: SNP data from the OsNR2 genomic sequences of 51 japonica rice varieties.

File Name: Supplementary Data 3.
Description: SNP data from the OsNR2 genomic sequences of 148 indica rice varieties.

File Name: Supplementary Data 4.
Description: SNP data from the OsNR2 genomic sequences of 23 wild rice accessions.
